# Pre-diabetes and Diabetes in Heart Transplant Recipients: Epidemiological Insights from a Decade-Long Study

**DOI:** 10.5812/ijem-168325

**Published:** 2026-06-22

**Authors:** Sanaz Soleimani, Fatemeh Kazemi Hasnijeh, Maryam Vasheghani, Farah Naghashzadeh, Zargham Hossein Ahmadi, Babak Sharif Kashani, Fatemeh Nouri, Niloufar Alizadeh Kolahdozi, Shadi Shafaghi

**Affiliations:** 1 Lung Transplantation Research Center (LTRC), National Research Institute of Tuberculosis and Lung Diseases (NRITLD), Shahid Beheshti University of Medical Sciences, Tehran, Iran; 2 School of Medicine, Shahid Beheshti University of Medical Sciences, Tehran, Iran; 3 Chronic Respiratory Diseases Research Center (CRDRC), National Research Institute of Tuberculosis and Lung Diseases (NRITLD), Shahid Beheshti University of Medical Sciences, Tehran, Iran; 4 Tobacco Prevention and Control Research Center, National Research Institute of Tuberculosis and Lun Diseases (NRITLD), Shahid Beheshti University of Medical Sciences, Tehran, Iran; 5 Department of Biostatistics, National Research Institute of Tuberculosis and Lung Disease (NRITLD), Shahid Beheshti University of Medical Sciences, Tehran, Iran

**Keywords:** Prediabetes, Diabetes, Heart Transplant, Epidemiology, Treatment Outcome

## Abstract

**Background:**

Post-transplant diabetes mellitus (PTDM) is a serious complication after heart transplantation, with reported incidence rates ranging from 2% to 53% among solid organ transplant recipients. Comprehensive epidemiological data on prediabetes (Pre-DM) and diabetes mellitus (DM) in heart transplant populations remain limited, particularly in Middle Eastern populations.

**Objectives:**

This decade-long study investigated the prevalence of DM and Pre-DM among heart transplant recipients and evaluated selected intraoperative risk factors.

**Methods:**

This retrospective cohort study included 250 heart transplant recipients who received post-transplant care at Dr. Masih Daneshvari Hospital, Tehran, Iran, between 2010 and 2020. Overall, 244 patients were included in the final analysis. Data included anthropometric measurements, cardiovascular parameters, and metabolic profiles. Pre-DM and DM were diagnosed according to the American Diabetes Association criteria. Statistical analyses were performed using SPSS version 26.

**Results:**

The study population comprised 192 males (78.7%) and 52 females (21.3%), with a mean age of 38.46 ± 12.69 years. At transplantation, 36.5% of recipients had established DM, 29.1% had Pre-DM, and 34.4% had normal glucose metabolism. Among patients with DM, 58.4% (52 of 89) were unaware of their diagnosis before transplantation. New-onset PTDM occurred in 6.8% of previously normoglycemic patients by 6 months. The prevalence of hypertension differed markedly by glucose status (P < 0.001): 100% of the normal group had normal blood pressure, 97.2% of patients with Pre-DM had stage 1 hypertension, and 87.6% of patients with DM had stage 2 hypertension. Dyslipidemia, particularly low high-density lipoprotein (HDL), was present in all groups. Pre-transplant hyperglycemia was associated with a numerically higher, but nonsignificant, mortality risk (hazard ratio [HR] = 1.371; 95% CI, 0.891 - 2.132; P = 0.162), which reversed after age adjustment (HR = 0.676; P = 0.083), consistent with negative confounding.

**Conclusions:**

Pre-transplant metabolic dysfunction was highly prevalent, and 58.4% of diabetic recipients were unaware of their diagnosis. Although the association with mortality was nonsignificant and reversed after adjustment for age, these findings underscore the need for systematic pre-transplant metabolic screening, post-transplant glucose monitoring, and multidisciplinary care in heart transplant recipients.

## 1. Background

The development of diabetes mellitus (DM) is a serious complication of heart transplantation and has substantial effects on patient outcomes and graft survival (1). The prevalence of post-transplant diabetes mellitus (PTDM) among solid organ transplant recipients ranges from 2% to 53%. Heart transplant recipients have higher rates, sometimes exceeding 30% within the first post-transplant year, depending on patient-related risk factors and diabetogenic immunosuppressive therapies (2, 3).

The pathogenesis of PTDM is multifactorial and is driven primarily by immunosuppressive agents, particularly corticosteroids and calcineurin inhibitors, which impair insulin secretion and promote peripheral insulin resistance (4, 5). The transplant procedure itself triggers inflammatory cascades that may exacerbate metabolic dysfunction and contribute to a bidirectional relationship between inflammation and glucose metabolism (6).

The clinical consequences extend beyond glycemic control. Transplant recipients with DM are at increased risk of infections and opportunistic infections because of impaired immune function (7, 8). DM also significantly increases the likelihood of chronic allograft rejection, with studies indicating a 2- to 3-fold increase in graft failure rates among patients with DM (9, 10). Additional contributors include the type of immunosuppressive regimen and genetic predisposition (11, 12). These factors highlight the importance of early diagnosis and effective treatment.

## 2. Objectives

This decade-long study investigated the prevalence of DM and pre-diabetes (Pre-DM) among heart transplant recipients and evaluated selected risk factors present at the time of transplantation.

## 3. Methods

### 3.1. Study Design and Setting

This retrospective cohort study enrolled patients at the time of heart transplantation, with pre-transplant glycemic status documented at baseline in 2010. All participants were followed through scheduled outpatient clinic visits at Dr. Masih Daneshvari Hospital at 1 week, 1 month, 6 months, and 12 months after transplantation and annually thereafter until 2020. Clinical, metabolic, and survival outcomes were extracted from medical records at each follow-up visit. Data were analyzed using SPSS version 26.

### 3.2. Data Collection

Anthropometric measurements included height, measured using a metal stadiometer with 0.5-cm accuracy, and weight, recorded to the nearest 0.1 kg using Seca scales. Body mass index (BMI) was calculated as weight (kg)/height^2^ (m^2^). Blood pressure was measured using a standard mercury sphygmomanometer after a rest period. Detailed metabolic profiles were extracted from patient medical records before transplant surgery and during follow-up visits. Glycemic control was assessed using fasting plasma glucose (FPG), 2-hour postprandial glucose (2HPP), and hemoglobin A1c (HbA1c), along with a complete lipid profile, including total cholesterol, low-density lipoprotein (LDL), HDL, and triglycerides.

### 3.3. Inclusion and Exclusion Criteria

Patients were included if they received follow-up care at our center and had complete pre- and post-transplant medical records, including metabolic parameters from the last pre-transplant admission. Patients who declined to provide written informed consent or had incomplete records were excluded.

### 3.4. Diagnostic Criteria

Diabetes mellitus was defined according to the American Diabetes Association 2020 criteria (13) as any of the following: FPG ≥ 126 mg/dL, 2-hour plasma glucose ≥ 200 mg/dL during a 75-g oral glucose tolerance test, or HbA1c ≥ 6.5%. Pre-DM was defined as FPG of 100 - 125 mg/dL (impaired fasting glucose), 2-hour plasma glucose of 140 - 199 mg/dL (impaired glucose tolerance), or HbA1c of 5.7% - 6.4%. Diagnoses were established using any combination of FPG, 2-hour plasma glucose, and HbA1c, where available. Hypertension was diagnosed according to the 2020 hypertension guideline criteria (14). World Health Organization criteria were used to define obesity.

Biosystem kits (S.A. Costa Brava, Spain) were used to assess serum lipid profiles using an autoanalyzer (Biosystem A25, Spain). The devices were calibrated daily.

### 3.5. Ethical Considerations

This study adhered to the Declaration of Helsinki. Written informed consent was obtained from all participants. The study protocol was approved by the Institutional Review Board of Iran University of Medical Sciences under the ethical approval code IR.SBMU.MSP.REC.1398.381.

## 4. Results

### 4.1. Study Population Characteristics

**Figure 1.**
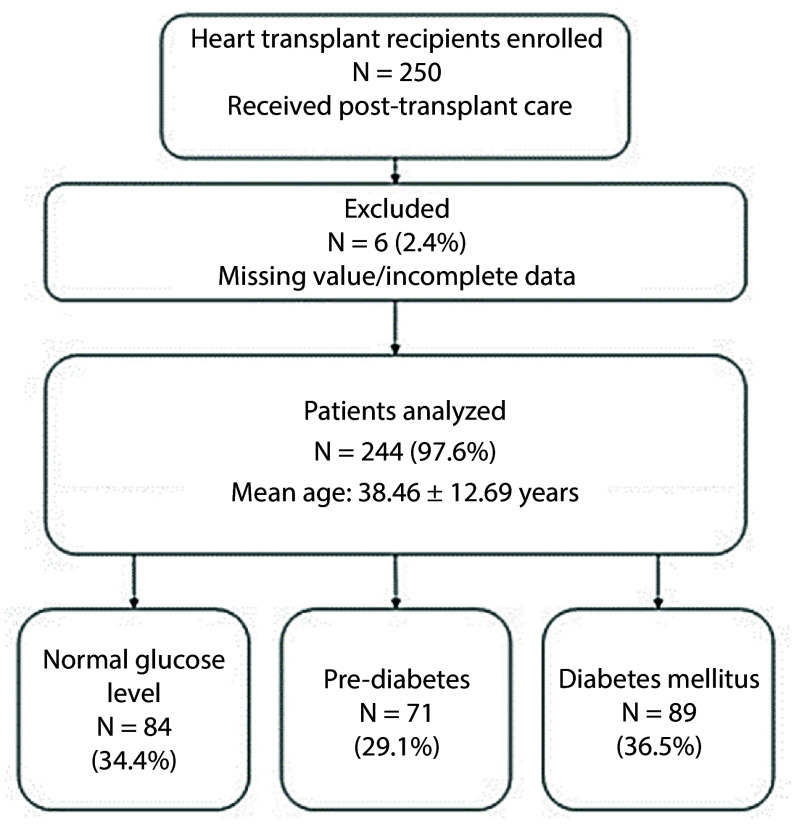
Study flowchart showing patient selection and glycemic status distribution among heart transplant recipients.

Among 250 heart transplant recipients, 244 (97.6%) were analyzed; 6 were excluded because of missing data. The study population comprised 192 males (78.7%) and 52 females (21.3%), with a mean age of 38.46 ± 12.69 years (Figure 1).

### 4.2. Prevalence of Diabetes Mellitus and Pre-Diabetes

The prevalence of glucose metabolism disorders was substantial in this cohort (Table 1). Among the analyzed patients, 84 (34.4%) had normal glucose metabolism, 71 (29.1%) had Pre-DM, and 89 (36.5%) had established DM at the time of transplantation. A family history of DM showed a nonsignificant increasing trend across the normal, Pre-DM, and DM groups (26.8%, 32.9%, and 42.5%, respectively; χ^2^ = 4.709; P = 0.095). Sex distribution (χ^2^ = 0.127; P = 0.939) and BMI categories (χ^2^ = 14.897; P = 0.061) were not significantly associated with glucose status.

**Table 1.** Baseline Characteristics of Heart Transplant Recipients Classified by Pre-Transplant Glucose Status ^a^

Variables	Normal	Pre-DM	DM	Total	Chi-Square	P-Value
**Gender**					0.127	0.939
Male	66 (78.6)	55 (77.5)	71 (79.8)	192 (78.7)		
**Family history of DM**					4.709	0.095
Positive	22 (26.8)	23 (32.9)	37 (42.5)	82 (34.3)		
**Blood pressure**					417.487	< 0.001
Normal	84 (100.0)	2 (2.8)	8 (9.0)	94 (38.5)		
Stage 1	0 (0.0)	69 (97.2)	3 (3.4)	72 (29.5)		
Stage 2	0 (0.0)	0 (0.0)	78 (87.6)	78 (32.0)		
**BMI**					14.897	0.061
Underweight	4 (7.5)	5 (8.9)	5 (6.8)	14 (7.7)		
Normal	30 (56.6)	33 (58.9)	43 (58.9)	106 (58.2)		
Overweight	14 (26.4)	10 (17.9)	22 (30.1)	46 (25.3)		
Obesity 1	5 (9.4)	3 (5.4)	3 (4.1)	11 (6.0)		
Obesity 2	0 (0.0)	5 (8.9)	0 (0.0)	5 (2.7)		

^a^ Values are expressed as No. (%). Type of treatment was reported only for the 30 patients with DM who had documented antidiabetic therapy at the last pre-transplant admission; the remaining 59 patients with DM had no recorded antidiabetic treatment. Nine patients in the normal group and 13 in the Pre-DM group received insulin during hospitalization for acute glycemic management (perioperative protocols and stress hyperglycemia), not as antidiabetic therapy; therefore, they were correctly classified in their respective glycemic groups.

Among the analyzed patients, 84 (34.4%) had normal glucose metabolism, 71 (29.1%) had Pre-DM, and 89 (36.5%) had established DM at transplantation. Of the 89 patients with pre-transplant DM, 52 (58.4%) had no prior documented diagnosis or antidiabetic therapy recorded in their medical records.

Glucose metabolism changed substantially during the early post-transplant period (Table 2). Among 71 of 84 patients with normal glucose metabolism who had available 1-month follow-up data, 37 (52.1%) developed new-onset DM; among 53 of 71 patients with Pre-DM, 34 (64.2%) progressed to DM; and among 82 of 89 patients with DM, 55 (67.1%) remained diabetic. A significant linear trend confirmed worsening 1-month glycemic status with a higher pre-transplant glucose category (P = 0.032; Table 2). By 6 months, persistent new-onset PTDM among patients without pre-existing DM was 6.8% (n = 17/155), consistent with resolution of hyperglycemia after immunosuppression tapering.

**Table 2.** Glycemic Status at 30 Days Post-Transplantation According to Pre-Transplant Glycemic Category ^a^

Pre-Heart Transplantation Status	Normal Glucose	Pre-DM	DM	Odds Ratio (95% CI)	P-Value	P-Value for Linear Trend
**Normal (n = 84)**	20 (28.2)	14 (19.7)	37 (52.1)	1.00	-	0.032 ^b^
**Pre-DM (n = 71)**	12 (22.6)	7 (13.2)	34 (64.2)	1.644 (0.793 - 3.410)	0.181	
**DM (n = 89)**	12 (14.6)	15 (18.3)	55 (67.1)	1.872 (0.972 - 3.603)	0.061	

^a^ Values are expressed as No. (%) unless otherwise indicated. Abbreviation: CI, confidence interval.

^b^ Linear-by-linear association.

### 4.3. Glycemic Control and Metabolic Parameters

Baseline metabolic and clinical characteristics stratified by pre-transplant glycemic status are presented in Table 3. Age differed significantly across groups (Kruskal-Wallis H = 12.939; P = 0.002), with patients with DM being significantly older than those in the normal group on post hoc analysis (Mann-Whitney test, P < 0.001 after *Bonferroni correction*). No significant difference was observed between the normal and Pre-DM groups (P = 0.169) or between the Pre-DM and DM groups (P = 0.034) after *Bonferroni correction* (adjusted threshold, P < 0.017).

**Table 3.** Baseline Demographic and Metabolic Characteristics of Heart Transplant Recipients Stratified by Pre-Transplant Glycemic Status ^a^

Variables	Total	Normal	Pre-DM	DM	P-Value
**Age (y)**	38.46 ± 12.69	35.07 ± 12.77	37.93 ± 12.31	42.09 ± 12.06	0.002 ^b^
**Length of initial admission after transplant (d)**	11.87 ± 1.94	11.25 ± 1.57	12.1 ± 1.86	12.35 ± 2.17	0.323 ^b^
**BMI (kg/m^2^)**	24.15 ± 4.54	24.19 ± 4.45	24.34 ± 5.67	23.99 ± 3.58	0.911
**HbA1c (%)**	7.69 ± 2.53	4.80 ± 0.70	5.84 ± 0.80	8.57 ± 2.45	< 0.001 ^b^
**HDL (mg/dL)**	33.15 ± 15.16	33.05 ± 16.54	32.68 ± 14.20	33.63 ± 14.66	0.455 ^b^
**TG (mg/dL)**	120.24 ± 113.32	102.17 ± 53.98	100.63 ± 36.49	154.14 ± 174.81	0.077 ^b^
**LDL (mg/dL)**	82.60 ± 33.97	79.73 ± 31.10	81.67 ± 33.14	86.35 ± 37.41	0.458
**Total cholesterol (mg/dL)**	140.89 ± 48.92	134.32 ± 42.46	135.07 ± 40.11	152.16 ± 58.96	0.032 ^c^

^a^ Values are expressed as mean ± SD.

^b^ Kruskal-Wallis test.

^c^ One-way analysis of variance.

Glycemic control varied markedly across groups, as reflected by HbA1c levels: 4.80% ± 0.70% in the normal group, 5.84% ± 0.80% in the Pre-DM group, and 8.57% ± 2.45% in the DM group (P < 0.001). BMI and length of initial admission after transplantation did not differ significantly across groups (P = 0.911 and P = 0.323, respectively).

Dyslipidemia was observed across all groups (Table 3). Total cholesterol was significantly higher in the DM group than in the normal group (152.16 ± 58.96 vs 134.32 ± 42.46 mg/dL; P = 0.032). HDL was uniformly low across all groups (overall mean, 33.15 ± 15.16 mg/dL; P = 0.455), and triglycerides were highest in the DM group (154.14 ± 174.81 mg/dL; P = 0.077).

### 4.4. Risk Factor Analysis

Chi-square analysis indicated significant associations between glucose status and clinical variables (Table 1). Blood pressure categories showed the strongest association with glucose status (χ^2^ = 417.487; P < 0.001): 100% of the normal group had normal blood pressure, 97.2% of patients with Pre-DM had stage 1 hypertension, and 87.6% of patients with DM had stage 2 hypertension.

### 4.5. Survival Analysis

The overall mean post-transplant follow-up duration was 96.8 months (95% CI, 85.9 - 107.7 months). By glycemic group, mean follow-up was 76.8 months in the normal glucose group, 102.0 months in the Pre-DM group, and 100.4 months in the DM group. Median survival did not reach 50%. Cumulative survival was 56.4% in the normal group and 64.6% in the Pre-DM group, reflecting favorable long-term survival.

Pre-transplant hyperglycemia was associated with a higher, but nonsignificant, mortality risk (HR = 1.371; 95% CI, 0.891 - 2.132; P = 0.162). Two additional multivariate Cox models were constructed to assess confounding by age and BMI. After adjustment for age (model 2), the HR reversed to 0.676 (95% CI, 0.435 - 1.052; P = 0.083), indicating negative confounding by age. After further adjustment for BMI (model 3; n = 180), the HR was 0.722 (95% CI, 0.413 - 1.260; P = 0.251); neither age nor BMI reached significance in this model. Crude and adjusted HRs are presented in Table 4.

**Table 4.** Crude and Adjusted Hazard Ratios for All-Cause Post-Transplant Mortality by Pre-transplant Glycemic Status ^a^

Covariate	Model 1: Crude HR (95% CI)	Model 1 P-Value	Model 2: Age-Adjusted HR (95% CI)	Model 2 P-Value	Model 3: Age- and BMI-Adjusted HR (95% CI)	Model 3 P-Value
**Hyperglycemia**	1.371 (0.891 - 2.132)	0.162	0.676 (0.435 - 1.052)	0.083	0.722 (0.413 - 1.260)	0.251
**Age**	-	-	1.012 (0.994 - 1.030)	0.202	1.010 (0.986 - 1.034)	0.427
**BMI**	-	-	-	-	1.022 (0.962 - 1.086)	0.477

^a^ Abbreviations: HR, hazard ratio; CI, confidence interval. Cox proportional hazards regression. Outcome: all-cause mortality (event = death; censoring = alive at last follow-up or lost to follow-up). The reversal of the hyperglycemia HR between model 1 and models 2 and 3 reflects negative confounding by age.

**Figure 2.**
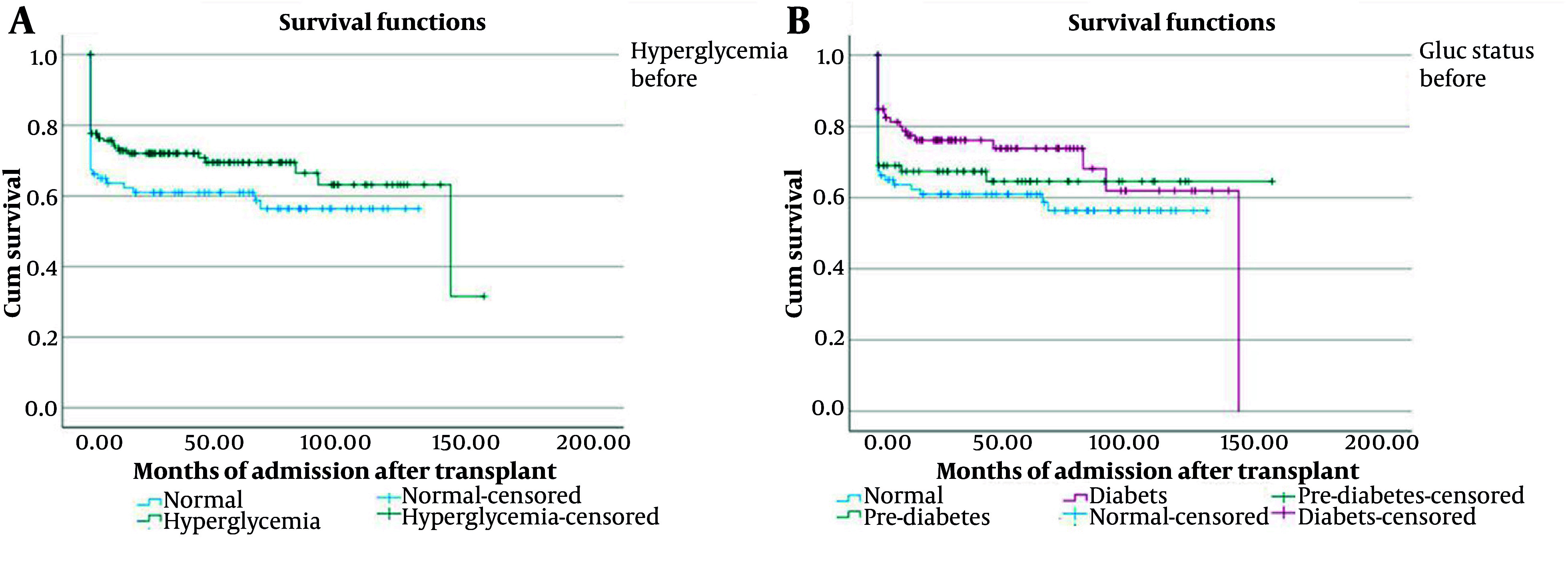
Kaplan-Meier survival curves for all-cause post-transplant mortality among heart transplant recipients. A, Comparison of recipients with normal pre-transplant glucose metabolism versus pre-transplant hyperglycemia; B, stratification by specific pre-transplant glycemic category.

Survival function analysis demonstrated separation between the normal and hyperglycemic groups throughout follow-up of up to 155.9 months (Figure 2). The log-rank comparison across all three glycemic groups was nonsignificant (χ^2^ = 3.182; df = 2; P = 0.204).

## 5. Discussion

This retrospective cohort study provides comprehensive epidemiological data on pre-transplant glucose metabolism disorders among Iranian heart transplant recipients, a region with a high DM burden (15).

Nearly two-thirds of recipients had established DM or Pre-DM at transplantation, a prevalence at the upper end of internationally reported ranges. Published literature and international consensus reports indicate a PTDM prevalence of 20% - 40% among heart transplant recipients, with most cases occurring within the first post-transplant year (16-18).

The high rate of undiagnosed pre-transplant DM (58.4%) and the rapid progression to hyperglycemia observed within the first month after transplantation have direct implications for patients’ self-management capacity. Khalooei and Hasheminejad demonstrated in Iranian patients with type 2 diabetes that diabetes management self-efficacy is suboptimal, with higher self-efficacy independently associated with better medication adherence and improved glycemic control (19).

The lower 6-month PTDM rate (6.8%) compared with published 1-year rates (15% - 30%) reflects the shorter observation window and our methodological reliance on FPG rather than HbA1c, which is unreliable perioperatively because cardiac recipients often receive frequent blood transfusions (20, 21). The cohort’s young mean age (38.46 ± 12.69 years) and male predominance (78.7%) reflect regional patterns. DM prevalence increases progressively with age, consistent with age being an established PTDM risk factor (22, 23).

The rapid progression to DM in 52.1% of previously normoglycemic patients within the first month underscores the acute diabetogenic burden of high-dose induction immunosuppression. Structured educational programs that include face-to-face training and remote follow-up have reduced hospitalization frequency and duration among cardiac patients with metabolic dysfunction (24).

The significant association between glucose metabolism disorders and hypertension (χ^2^ = 417.487; P < 0.001) is consistent with the reported hypertension prevalence of 50% - 95% among heart transplant patients, largely attributable to calcineurin inhibitor use (25).

Uniformly low HDL levels across all groups (mean, 33.15 mg/dL) reflect an atherogenic lipid phenotype attributable to the pre-existing cardiovascular disease burden and immunosuppressive effects, underscoring the need for active lipid management regardless of glycemic category (26).

The inversion of the crude HR from 1.371 to 0.676 after age adjustment is consistent with negative confounding by age. Patients with DM in this cohort were significantly older than normoglycemic recipients, and older recipient age is a well-established determinant of post-transplant mortality (27).

Several limitations should be acknowledged. First, some patients residing outside Tehran returned to regional medical centers for routine post-transplant follow-up, and COVID-19-related disruptions to outpatient care in 2020 further contributed to incomplete follow-up. Second, incomplete data on immunosuppressive regimens and transplant indications limited adjustment for established PTDM risk factors. Future studies should adopt a multicenter prospective design with standardized follow-up protocols and a multidisciplinary approach to improve the generalizability of findings across diverse transplant populations.

### 5.1. Conclusions

This study found a high prevalence of pre-transplant metabolic dysfunction (36.5% DM and 29.1% Pre-DM), with a 6-month PTDM incidence of 6.8%. The marked 1-month increase in DM confirms the acute diabetogenic impact of perioperative immunosuppression. These findings support the critical role of metabolic screening before transplantation, glucose monitoring after transplantation, and coordinated multidisciplinary care for comprehensive patient management.

## Data Availability

The dataset presented in the study is available on request from the corresponding author during submission or after publication.
